# Hexane-1,6-diammonium bis­(pyridine-2-carboxyl­ate)

**DOI:** 10.1107/S1600536809019424

**Published:** 2009-05-29

**Authors:** Nam-Ho Kim, Kwang Ha

**Affiliations:** a School of Applied Chemical Engineering, Research Institute of Catalysis, Chonnam National University, Gwangju 500-757, Republic of Korea

## Abstract

The title compound, C_6_H_18_N_2_^2+^·2C_6_H_4_NO_2_^−^, consists of a doubly protonated hexa­methyl­enediammonium dication and two pyridine-2-carboxyl­ate anions. These ions inter­act by means of inter­molecular N—H⋯O and N—H⋯N hydrogen bonds to form a two-dimensional array. The carboxyl­ate groups of the anions appear to be delocalized on the basis of the C—O bond lengths.

## Related literature

For the crystal structures of (C_6_H_18_N_2_)*X*_2_ (*X* = Cl, Br or I), see: Binnie & Robertson (1949*a* ▶,*b* ▶); Borkakoti *et al.* (1978 ▶); van Blerk & Kruger (2008 ▶). For details of some other hexane-1,6-diammonium compounds, see: Phan Thanh *et al.* (2000 ▶); Mousdis *et al.* (2000 ▶); Rakovský *et al.* (2002 ▶); Dammak *et al.* (2007 ▶); Sun *et al.* (2007 ▶); Yang *et al.* (2007 ▶); Wilkinson & Harrison (2007 ▶); Wang & Wei (2007 ▶). For the structure of pyridine-2-carboxylic acid, see: Hamazaki *et al.* (1998 ▶).
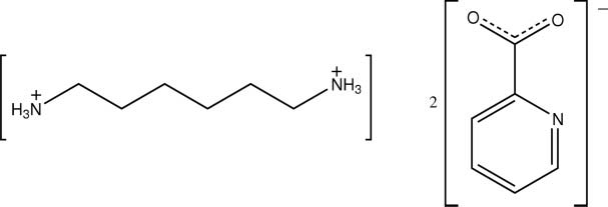


## Experimental

### 

#### Crystal data

C_6_H_18_N_2_^2+^·2C_6_H_4_NO_2_^−^
*M* *_r_* = 362.43
Monoclinic, 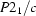

*a* = 9.8182 (7) Å
*b* = 9.1569 (7) Å
*c* = 21.6423 (17) Å
β = 99.038 (2)°
*V* = 1921.6 (3) Å^3^
*Z* = 4
Mo *K*α radiation
μ = 0.09 mm^−1^
*T* = 296 K
0.33 × 0.25 × 0.18 mm

#### Data collection

Bruker SMART 1000 CCD diffractometer
Absorption correction: multi-scan (*SADABS*; Bruker, 2000 ▶) *T*_min_ = 0.685, *T*_max_ = 0.984
13964 measured reflections
4752 independent reflections
1740 reflections with *I* > 2σ(*I*)
*R*_int_ = 0.089

#### Refinement

*R*[*F* ^2^ > 2σ(*F* ^2^)] = 0.057
*wR*(*F* ^2^) = 0.124
*S* = 0.93
4752 reflections
339 parameters
All H-atom parameters refined
Δρ_max_ = 0.15 e Å^−3^
Δρ_min_ = −0.15 e Å^−3^

### 

Data collection: *SMART* (Bruker, 2000 ▶); cell refinement: *SAINT* (Bruker, 2000 ▶); data reduction: *SAINT*; program(s) used to solve structure: *SHELXS97* (Sheldrick, 2008 ▶); program(s) used to refine structure: *SHELXL97* (Sheldrick, 2008 ▶); molecular graphics: *ORTEP-3* (Farrugia, 1997 ▶) and *PLATON* (Spek, 2009 ▶); software used to prepare material for publication: *SHELXL97*.

## Supplementary Material

Crystal structure: contains datablocks global, I. DOI: 10.1107/S1600536809019424/tk2459sup1.cif

Structure factors: contains datablocks I. DOI: 10.1107/S1600536809019424/tk2459Isup2.hkl

Additional supplementary materials:  crystallographic information; 3D view; checkCIF report

## Figures and Tables

**Table 1:** Hydrogen-bond geometry (Å, °)

*D*—H⋯*A*	*D*—H	H⋯*A*	*D*⋯*A*	*D*—H⋯*A*
N3—H3*A*⋯O4	1.07 (3)	1.70 (3)	2.747 (3)	165 (3)
N3—H3*B*⋯O1^i^	0.96 (3)	2.29 (3)	3.088 (3)	140 (2)
N3—H3*B*⋯N1^i^	0.96 (3)	2.15 (3)	2.962 (3)	142 (2)
N3—H3*C*⋯O1^ii^	0.92 (3)	1.91 (3)	2.835 (3)	177 (3)
N4—H4*A*⋯O3^iii^	0.97 (3)	2.27 (3)	3.064 (3)	139 (2)
N4—H4*A*⋯N2^iii^	0.97 (3)	2.12 (3)	2.963 (3)	144 (2)
N4—H4*B*⋯O3^iv^	1.07 (3)	1.67 (3)	2.740 (3)	175 (3)
N4—H4*C*⋯O2^v^	1.05 (3)	1.70 (4)	2.754 (3)	179 (3)
C1—H1⋯O4^vi^	1.02 (3)	2.45 (3)	3.328 (4)	145 (2)
C16—H16*B*⋯O3^iv^	1.01 (3)	2.58 (3)	3.426 (4)	140.8 (18)

Symmetry codes: (i) 
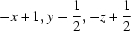
; (ii) 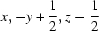; (iii) 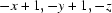; (iv) 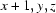; (v) 
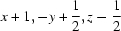
; (vi) 
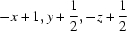
.

**Fig. 1.**
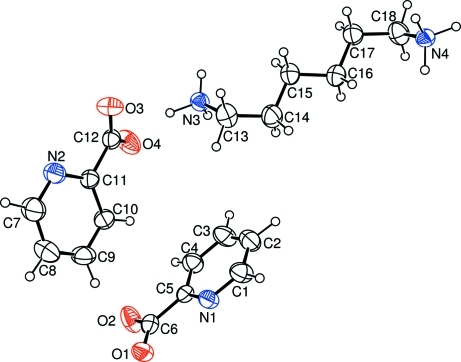
The molecular structures of the components (I), with displacement ellipsoids drawn at the 30% probability level for non-H atoms.

**Fig. 2.**
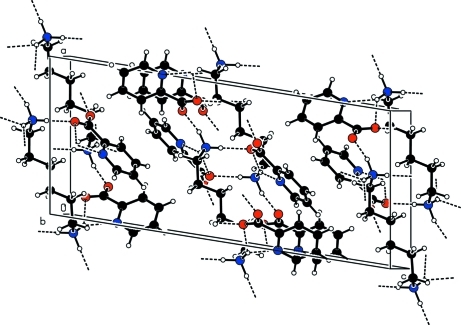
View of the unit-cell contents for (I). Hydrogen-bond interactions are drawn with dashed lines.

## supplementary crystallographic information

### Comment

The title compound, C_6_H_18_N_2_^2+^.2C_6_H_4_NO_2_^-^, consists of a doubly protonated hexamethylenediammonium dication and two pyridinecarboxylate anions (Fig. 1). The carboxylate groups of the anions appear to be delocalized on the basis of the C—O bond lengths (C—O: 1.241 (3)–1.247 (3) Å). The N3—C13—C14—C15 and C16—C17—C18—N4 torsion angles [64.9 (4)° and -66.6 (4)°, respectively] display the *gauche* conformation for the two groups within the dication, whereas C13—C14—C15—C16, C14—C15—C16—C17 and C15—C16—C17—C18 atoms show the anti conformation [their torsion angles lie in the range of 174.6 (3)°–177.3 (3)°]. In the crystal, the component ions interact by means of many intermolecular N—H···O and N—H···N hydrogen bonds and C—H···O contacts to form a 2-D array (Table 1 and Fig. 2).

### Experimental

A solution of 1,6-diaminohexane (0.200 g, 1.721 mmol) and pyridine-2-carboxylic acid (1.180 g, 8.606 mmol) in H_2_O (20 ml) was stirred for 2 h at 333 K. The solvent was removed under vacuum and the residue was washed with acetone to give a white powder (0.5972 g). Crystals were obtained by slow evaporation from an ethanol solution.

### Refinement

All H atoms were located from Fourier difference maps and refined isotropically; C—H = 0.96 (3)–1.13 (3) Å and N—H = 0.92 (3)–1.07 (3) Å.

### Crystal data

**Table:**

C_6_H_18_N_2_^2+^·2C_6_H_4_NO_2_^−^	*F*(000) = 776
*M**_r_* = 362.43	*D*_x_ = 1.253 Mg m^−^^3^
Monoclinic, *P*2_1_/*c*	Mo *K*α radiation, λ = 0.71073 Å
Hall symbol: -P 2ybc	Cell parameters from 1472 reflections
*a* = 9.8182 (7) Å	θ = 2.6–24.0°
*b* = 9.1569 (7) Å	µ = 0.09 mm^−^^1^
*c* = 21.6423 (17) Å	*T* = 296 K
β = 99.038 (2)°	Block, colourless
*V* = 1921.6 (3) Å^3^	0.33 × 0.25 × 0.18 mm
*Z* = 4	

### Data collection

**Table:**

Bruker SMART 1000 CCD diffractometer	4752 independent reflections
Radiation source: fine-focus sealed tube	1740 reflections with *I* > 2σ(*I*)
graphite	*R*_int_ = 0.089
φ and ω scans	θ_max_ = 28.3°, θ_min_ = 1.9°
Absorption correction: multi-scan (*SADABS*; Bruker, 2000)	*h* = −13→13
*T*_min_ = 0.685, *T*_max_ = 0.984	*k* = −12→12
13964 measured reflections	*l* = −21→28

### Refinement

**Table:**

Refinement on *F*^2^	Primary atom site location: structure-invariant direct methods
Least-squares matrix: full	Secondary atom site location: difference Fourier map
*R*[*F*^2^ > 2σ(*F*^2^)] = 0.057	Hydrogen site location: inferred from neighbouring sites
*wR*(*F*^2^) = 0.124	All H-atom parameters refined
*S* = 0.93	*w* = 1/[σ^2^(*F*_o_^2^) + (0.0318*P*)^2^] where *P* = (*F*_o_^2^ + 2*F*_c_^2^)/3
4752 reflections	(Δ/σ)_max_ < 0.001
339 parameters	Δρ_max_ = 0.15 e Å^−^^3^
0 restraints	Δρ_min_ = −0.15 e Å^−^^3^

### Special details

**Table:**

Geometry. All e.s.d.'s (except the e.s.d. in the dihedral angle between two l.s. planes) are estimated using the full covariance matrix. The cell e.s.d.'s are taken into account individually in the estimation of e.s.d.'s in distances, angles and torsion angles; correlations between e.s.d.'s in cell parameters are only used when they are defined by crystal symmetry. An approximate (isotropic) treatment of cell e.s.d.'s is used for estimating e.s.d.'s involving l.s. planes.
Refinement. Refinement of *F*^2^ against ALL reflections. The weighted *R*-factor *wR* and goodness of fit *S* are based on *F*^2^, conventional *R*-factors *R* are based on *F*, with *F* set to zero for negative *F*^2^. The threshold expression of *F*^2^ > σ(*F*^2^) is used only for calculating *R*-factors(gt) *etc*. and is not relevant to the choice of reflections for refinement. *R*-factors based on *F*^2^ are statistically about twice as large as those based on *F*, and *R*- factors based on ALL data will be even larger.

### Fractional atomic coordinates and isotropic or equivalent isotropic displacement parameters (Å^2^)

**Table:**

	*x*	*y*	*z*	*U*_iso_*/*U*_eq_	
O1	0.39197 (19)	0.3983 (2)	0.43294 (9)	0.0607 (6)	
O2	0.3440 (2)	0.1705 (2)	0.40142 (11)	0.0809 (7)	
N1	0.5671 (2)	0.4287 (2)	0.34905 (10)	0.0514 (6)	
C1	0.6534 (3)	0.4405 (4)	0.30699 (15)	0.0632 (9)	
H1	0.704 (3)	0.538 (3)	0.3085 (12)	0.078 (10)*	
C2	0.6711 (3)	0.3335 (4)	0.26461 (16)	0.0684 (9)	
H2	0.739 (3)	0.351 (3)	0.2336 (14)	0.100 (11)*	
C3	0.5975 (4)	0.2072 (4)	0.26545 (16)	0.0702 (10)	
H3	0.605 (3)	0.128 (3)	0.2356 (13)	0.080 (10)*	
C4	0.5110 (3)	0.1905 (3)	0.30952 (14)	0.0586 (8)	
H4	0.458 (2)	0.099 (3)	0.3141 (12)	0.061 (8)*	
C5	0.4980 (3)	0.3030 (3)	0.35030 (12)	0.0428 (6)	
C6	0.4034 (3)	0.2901 (3)	0.39927 (14)	0.0508 (7)	
O3	0.1328 (2)	0.4425 (2)	0.07130 (9)	0.0654 (6)	
O4	0.2162 (2)	0.2478 (2)	0.12601 (10)	0.0760 (7)	
N2	0.0026 (2)	0.5187 (3)	0.16852 (11)	0.0617 (7)	
C7	−0.0394 (4)	0.5760 (4)	0.21915 (18)	0.0749 (10)	
H7	−0.127 (3)	0.630 (3)	0.2088 (13)	0.084 (10)*	
C8	0.0265 (4)	0.5565 (4)	0.27899 (17)	0.0718 (10)	
H8	−0.010 (3)	0.603 (3)	0.3131 (14)	0.080 (10)*	
C9	0.1416 (4)	0.4708 (4)	0.28807 (15)	0.0653 (9)	
H9	0.195 (3)	0.456 (3)	0.3287 (12)	0.066 (9)*	
C10	0.1851 (3)	0.4064 (3)	0.23713 (14)	0.0545 (8)	
H10	0.268 (3)	0.344 (3)	0.2409 (13)	0.083 (10)*	
C11	0.1141 (3)	0.4333 (3)	0.17813 (13)	0.0443 (7)	
C12	0.1582 (3)	0.3693 (3)	0.12002 (14)	0.0479 (7)	
N3	0.4265 (3)	0.1748 (3)	0.06174 (13)	0.0505 (7)	
H3A	0.334 (3)	0.200 (3)	0.0800 (14)	0.114 (12)*	
H3B	0.467 (3)	0.090 (3)	0.0830 (15)	0.095 (12)*	
H3C	0.415 (3)	0.154 (3)	0.0194 (16)	0.092 (12)*	
N4	1.1032 (3)	0.3355 (3)	−0.04832 (13)	0.0487 (6)	
H4A	1.046 (3)	0.403 (3)	−0.0754 (12)	0.068 (10)*	
H4B	1.117 (3)	0.372 (3)	−0.0007 (16)	0.099 (11)*	
H4C	1.196 (4)	0.333 (3)	−0.0672 (15)	0.126 (13)*	
C13	0.5150 (3)	0.3063 (4)	0.07920 (18)	0.0655 (9)	
H13A	0.500 (3)	0.334 (3)	0.1259 (15)	0.101 (11)*	
H13B	0.475 (3)	0.388 (3)	0.0459 (14)	0.099 (11)*	
C14	0.6652 (4)	0.2789 (4)	0.07806 (17)	0.0676 (10)	
H14A	0.718 (3)	0.372 (3)	0.0970 (13)	0.086 (10)*	
H14B	0.697 (3)	0.194 (3)	0.1038 (14)	0.090 (12)*	
C15	0.6995 (3)	0.2449 (4)	0.01361 (16)	0.0600 (9)	
H15A	0.654 (3)	0.150 (3)	−0.0029 (13)	0.073 (9)*	
H15B	0.654 (3)	0.336 (3)	−0.0184 (14)	0.100 (11)*	
C16	0.8544 (3)	0.2304 (4)	0.01326 (16)	0.0578 (8)	
H16A	0.895 (3)	0.153 (3)	0.0455 (12)	0.072 (9)*	
H16B	0.898 (2)	0.328 (3)	0.0250 (12)	0.070 (9)*	
C17	0.8877 (3)	0.1893 (4)	−0.05053 (16)	0.0623 (9)	
H17A	0.841 (3)	0.261 (3)	−0.0839 (13)	0.075 (10)*	
H17B	0.851 (3)	0.083 (3)	−0.0641 (14)	0.103 (11)*	
C18	1.0393 (4)	0.1871 (4)	−0.05523 (19)	0.0660 (9)	
H18A	1.054 (3)	0.150 (3)	−0.1001 (15)	0.110 (12)*	
H18B	1.088 (3)	0.123 (3)	−0.0212 (15)	0.102 (13)*	

### Atomic displacement parameters (Å^2^)

**Table:**

	*U*^11^	*U*^22^	*U*^33^	*U*^12^	*U*^13^	*U*^23^
O1	0.0655 (13)	0.0674 (13)	0.0506 (12)	0.0062 (11)	0.0135 (11)	−0.0096 (11)
O2	0.0771 (15)	0.0718 (15)	0.1046 (19)	−0.0218 (12)	0.0482 (14)	−0.0166 (14)
N1	0.0577 (15)	0.0545 (15)	0.0435 (14)	−0.0030 (12)	0.0132 (12)	−0.0014 (12)
C1	0.077 (2)	0.062 (2)	0.053 (2)	−0.0093 (19)	0.0165 (19)	−0.0003 (19)
C2	0.069 (2)	0.079 (3)	0.062 (2)	−0.006 (2)	0.0240 (19)	−0.007 (2)
C3	0.083 (2)	0.071 (2)	0.061 (2)	0.008 (2)	0.026 (2)	−0.019 (2)
C4	0.065 (2)	0.0547 (19)	0.058 (2)	−0.0009 (17)	0.0143 (17)	−0.0086 (18)
C5	0.0387 (15)	0.0484 (16)	0.0397 (16)	0.0027 (14)	0.0009 (13)	0.0009 (15)
C6	0.0406 (17)	0.0568 (19)	0.0536 (19)	0.0032 (16)	0.0037 (15)	−0.0004 (17)
O3	0.0893 (16)	0.0646 (13)	0.0413 (12)	0.0046 (11)	0.0075 (11)	0.0000 (12)
O4	0.0864 (16)	0.0717 (14)	0.0775 (16)	0.0331 (13)	0.0361 (13)	0.0103 (13)
N2	0.0638 (17)	0.0723 (17)	0.0496 (16)	0.0235 (14)	0.0108 (14)	0.0054 (14)
C7	0.076 (3)	0.083 (3)	0.066 (2)	0.031 (2)	0.014 (2)	0.005 (2)
C8	0.088 (3)	0.073 (2)	0.060 (2)	0.010 (2)	0.029 (2)	−0.002 (2)
C9	0.077 (3)	0.077 (2)	0.041 (2)	−0.001 (2)	0.0073 (19)	0.007 (2)
C10	0.0522 (19)	0.062 (2)	0.0489 (19)	0.0088 (16)	0.0048 (17)	0.0112 (18)
C11	0.0437 (17)	0.0441 (16)	0.0447 (17)	0.0016 (14)	0.0055 (14)	0.0061 (15)
C12	0.0424 (17)	0.0504 (18)	0.0510 (19)	−0.0023 (14)	0.0073 (15)	0.0037 (16)
N3	0.0516 (16)	0.0521 (16)	0.0487 (17)	0.0030 (14)	0.0111 (14)	0.0051 (15)
N4	0.0480 (15)	0.0531 (16)	0.0445 (15)	0.0048 (13)	0.0060 (14)	0.0050 (14)
C13	0.066 (2)	0.055 (2)	0.079 (3)	−0.0051 (18)	0.021 (2)	−0.008 (2)
C14	0.063 (2)	0.070 (2)	0.072 (3)	−0.010 (2)	0.016 (2)	−0.013 (2)
C15	0.052 (2)	0.066 (2)	0.063 (2)	−0.0077 (18)	0.0128 (18)	0.000 (2)
C16	0.052 (2)	0.058 (2)	0.064 (2)	−0.0051 (17)	0.0086 (17)	0.0004 (19)
C17	0.059 (2)	0.069 (2)	0.061 (2)	−0.0086 (19)	0.0140 (18)	−0.008 (2)
C18	0.069 (2)	0.060 (2)	0.074 (3)	−0.0004 (19)	0.025 (2)	−0.010 (2)

### Geometric parameters (Å, °)

**Table:**

O1—C6	1.245 (3)	N3—C13	1.498 (4)
O2—C6	1.245 (3)	N3—H3A	1.07 (3)
N1—C5	1.339 (3)	N3—H3B	0.96 (3)
N1—C1	1.341 (3)	N3—H3C	0.92 (3)
C1—C2	1.371 (4)	N4—C18	1.495 (4)
C1—H1	1.02 (3)	N4—H4A	0.97 (3)
C2—C3	1.365 (4)	N4—H4B	1.07 (3)
C2—H2	1.03 (3)	N4—H4C	1.05 (3)
C3—C4	1.381 (4)	C13—C14	1.500 (4)
C3—H3	0.98 (3)	C13—H13A	1.08 (3)
C4—C5	1.375 (3)	C13—H13B	1.07 (3)
C4—H4	1.00 (2)	C14—C15	1.518 (4)
C5—C6	1.520 (3)	C14—H14A	1.05 (3)
O3—C12	1.241 (3)	C14—H14B	0.98 (3)
O4—C12	1.247 (3)	C15—C16	1.528 (4)
N2—C11	1.335 (3)	C15—H15A	1.01 (3)
N2—C7	1.337 (4)	C15—H15B	1.13 (3)
C7—C8	1.365 (4)	C16—C17	1.515 (4)
C7—H7	0.99 (3)	C16—H16A	1.03 (3)
C8—C9	1.365 (4)	C16—H16B	1.01 (3)
C8—H8	0.97 (3)	C17—C18	1.508 (4)
C9—C10	1.376 (4)	C17—H17A	1.03 (3)
C9—H9	0.96 (3)	C17—H17B	1.06 (3)
C10—C11	1.378 (4)	C18—H18A	1.06 (3)
C10—H10	0.99 (3)	C18—H18B	1.01 (3)
C11—C12	1.512 (4)		
			
C5—N1—C1	117.3 (3)	H3B—N3—H3C	107 (3)
N1—C1—C2	123.8 (3)	C18—N4—H4A	108.7 (15)
N1—C1—H1	114.0 (15)	C18—N4—H4B	111.5 (16)
C2—C1—H1	122.2 (15)	H4A—N4—H4B	111 (2)
C3—C2—C1	118.2 (3)	C18—N4—H4C	108.3 (18)
C3—C2—H2	122.6 (17)	H4A—N4—H4C	104 (2)
C1—C2—H2	119.1 (17)	H4B—N4—H4C	113 (2)
C2—C3—C4	119.1 (3)	N3—C13—C14	113.3 (3)
C2—C3—H3	121.3 (16)	N3—C13—H13A	105.3 (16)
C4—C3—H3	119.6 (16)	C14—C13—H13A	109.7 (16)
C5—C4—C3	119.3 (3)	N3—C13—H13B	104.9 (16)
C5—C4—H4	117.4 (15)	C14—C13—H13B	111.3 (16)
C3—C4—H4	123.3 (15)	H13A—C13—H13B	112 (2)
N1—C5—C4	122.2 (3)	C13—C14—C15	114.2 (3)
N1—C5—C6	116.6 (2)	C13—C14—H14A	106.5 (15)
C4—C5—C6	121.2 (3)	C15—C14—H14A	111.1 (15)
O2—C6—O1	126.3 (3)	C13—C14—H14B	110.6 (18)
O2—C6—C5	115.8 (3)	C15—C14—H14B	105.3 (18)
O1—C6—C5	117.9 (3)	H14A—C14—H14B	109 (2)
C11—N2—C7	116.9 (3)	C14—C15—C16	112.8 (3)
N2—C7—C8	124.4 (3)	C14—C15—H15A	110.5 (15)
N2—C7—H7	112.3 (17)	C16—C15—H15A	108.0 (15)
C8—C7—H7	123.2 (17)	C14—C15—H15B	106.8 (15)
C9—C8—C7	118.0 (3)	C16—C15—H15B	111.0 (14)
C9—C8—H8	122.6 (17)	H15A—C15—H15B	108 (2)
C7—C8—H8	119.4 (17)	C17—C16—C15	112.5 (3)
C8—C9—C10	119.0 (3)	C17—C16—H16A	109.2 (14)
C8—C9—H9	122.0 (16)	C15—C16—H16A	109.9 (14)
C10—C9—H9	118.9 (16)	C17—C16—H16B	107.8 (15)
C9—C10—C11	119.4 (3)	C15—C16—H16B	107.9 (14)
C9—C10—H10	122.4 (17)	H16A—C16—H16B	110 (2)
C11—C10—H10	118.2 (17)	C18—C17—C16	114.9 (3)
N2—C11—C10	122.2 (3)	C18—C17—H17A	107.2 (15)
N2—C11—C12	115.7 (3)	C16—C17—H17A	110.3 (15)
C10—C11—C12	122.2 (3)	C18—C17—H17B	105.3 (16)
O3—C12—O4	126.7 (3)	C16—C17—H17B	111.6 (16)
O3—C12—C11	116.8 (3)	H17A—C17—H17B	107 (2)
O4—C12—C11	116.5 (3)	N4—C18—C17	112.6 (3)
C13—N3—H3A	103.1 (16)	N4—C18—H18A	105.7 (17)
C13—N3—H3B	110.6 (18)	C17—C18—H18A	110.1 (17)
H3A—N3—H3B	108 (2)	N4—C18—H18B	108.5 (18)
C13—N3—H3C	113.2 (19)	C17—C18—H18B	108.7 (18)
H3A—N3—H3C	115 (3)	H18A—C18—H18B	111 (2)
			
C5—N1—C1—C2	2.0 (4)	C8—C9—C10—C11	1.9 (5)
N1—C1—C2—C3	−0.5 (5)	C7—N2—C11—C10	−1.1 (4)
C1—C2—C3—C4	−1.4 (5)	C7—N2—C11—C12	179.5 (3)
C2—C3—C4—C5	1.8 (5)	C9—C10—C11—N2	−0.9 (4)
C1—N1—C5—C4	−1.5 (4)	C9—C10—C11—C12	178.5 (3)
C1—N1—C5—C6	178.6 (2)	N2—C11—C12—O3	30.2 (3)
C3—C4—C5—N1	−0.3 (4)	C10—C11—C12—O3	−149.2 (3)
C3—C4—C5—C6	179.6 (3)	N2—C11—C12—O4	−149.6 (2)
N1—C5—C6—O2	−177.3 (3)	C10—C11—C12—O4	31.0 (4)
C4—C5—C6—O2	2.8 (4)	N3—C13—C14—C15	64.9 (4)
N1—C5—C6—O1	2.9 (4)	C13—C14—C15—C16	175.1 (3)
C4—C5—C6—O1	−177.0 (3)	C14—C15—C16—C17	177.3 (3)
C11—N2—C7—C8	2.2 (5)	C15—C16—C17—C18	174.6 (3)
N2—C7—C8—C9	−1.2 (6)	C16—C17—C18—N4	−66.6 (4)
C7—C8—C9—C10	−0.9 (5)		

### Hydrogen-bond geometry (Å, °)

**Table:**

*D*—H···*A*	*D*—H	H···*A*	*D*···*A*	*D*—H···*A*
N3—H3A···O4	1.07 (3)	1.70 (3)	2.747 (3)	165 (3)
N3—H3B···O1^i^	0.96 (3)	2.29 (3)	3.088 (3)	140 (2)
N3—H3B···N1^i^	0.96 (3)	2.15 (3)	2.962 (3)	142 (2)
N3—H3C···O1^ii^	0.92 (3)	1.91 (3)	2.835 (3)	177 (3)
N4—H4A···O3^iii^	0.97 (3)	2.27 (3)	3.064 (3)	139 (2)
N4—H4A···N2^iii^	0.97 (3)	2.12 (3)	2.963 (3)	144 (2)
N4—H4B···O3^iv^	1.07 (3)	1.67 (3)	2.740 (3)	175 (3)
N4—H4C···O2^v^	1.05 (3)	1.70 (4)	2.754 (3)	179 (3)
C1—H1···O4^vi^	1.02 (3)	2.45 (3)	3.328 (4)	145 (2)
C16—H16B···O3^iv^	1.01 (3)	2.58 (3)	3.426 (4)	140.8 (18)

Symmetry codes: (i) −*x*+1, *y*−1/2, −*z*+1/2; (ii) *x*, −*y*+1/2, *z*−1/2; (iii) −*x*+1, −*y*+1, −*z*; (iv) *x*+1, *y*, *z*; (v) *x*+1, −*y*+1/2, *z*−1/2; (vi) −*x*+1, *y*+1/2, −*z*+1/2.

## References

[bb1] Binnie, W. P. & Robertson, J. M. (1949*a*). *Acta Cryst.* **2**, 116–120.

[bb2] Binnie, W. P. & Robertson, J. M. (1949*b*). *Acta Cryst.* **2**, 180–188.

[bb3] Blerk, C. van & Kruger, G. J. (2008). *Acta Cryst.* C**64**, o537–o542. 10.1107/S0108270108022919 18838770

[bb4] Borkakoti, N., Lindley, P. F., Moss, D. S. & Palmer, R. A. (1978). *Acta Cryst.* B**34**, 3431–3433.

[bb5] Bruker (2000). *SADABS*, *SMART* and *SAINT* Bruker AXS Inc., Madison, Wisconsin, USA.

[bb6] Dammak, T., Fourati, N., Boughzala, H., Mlayah, A. & Abid, Y. (2007). *J. Lumin.* **127**, 404–408. 10.1016/j.saa.2006.05.018 16876465

[bb7] Farrugia, L. J. (1997). *J. Appl. Cryst.* **30**, 565.

[bb8] Hamazaki, H., Hosomi, H., Takeda, S., Kataoka, H. & Ohba, S. (1998). *Acta Cryst.* C**54** IUC9800049.

[bb9] Mousdis, G. A., Papavassiliou, G. C., Raptopoulou, C. P. & Terzis, A. (2000). *J. Mater. Chem.* **10**, 515–518.

[bb10] Phan Thanh, S., Renaudin, J. & Maisonneuve, V. (2000). *Solid State Sci.* **2**, 143–148.

[bb11] Rakovský, E., Žúrková, L. & Marek, J. (2002). *Monatsh. Chem.* **133**, 277–283.

[bb12] Sheldrick, G. M. (2008). *Acta Cryst.* A**64**, 112–122. 10.1107/S0108767307043930 18156677

[bb13] Spek, A. L. (2009). *Acta Cryst.* D**65**, 148–155. 10.1107/S090744490804362X PMC2631630 19171970

[bb14] Sun, D., Zhang, H., Zhang, J., Zheng, G., Yu, J. & Gao, S. (2007). *J. Solid State Chem.* **180**, 393–399.

[bb15] Wang, Z.-L. & Wei, L.-H. (2007). *Acta Cryst.* E**63**, o995–o996.

[bb16] Wilkinson, H. S. & Harrison, W. T. A. (2007). *Acta Cryst.* E**63**, m902–m904.

[bb17] Yang, S., Li, G., Tian, S., Liao, F., Xiong, M. & Lin, J. (2007). *J. Solid State Chem.* **180**, 2225–2232.

